# Spinal Cord Reperfusion Injury: Case Report, Review of the Literature, and Future Treatment Strategies

**DOI:** 10.7759/cureus.5279

**Published:** 2019-07-30

**Authors:** James G Wiginton, James Brazdzionis, Cyrus Mohrdar, Raed Sweiss, Shokry Lawandy

**Affiliations:** 1 Neurosurgery, Riverside University Health System Medical Center, Moreno Valley, USA; 2 Medicine, Western University of Health Sciences, Pomona, USA

**Keywords:** cervical spondylotic myelopathy, reperfusion, cervical myelopathy, cervical spondylosis, myelopathy, white cord syndrome, re-expansion, paralysis

## Abstract

A rare complication of cervical spine decompression is acute paralysis following the procedure. This neurologic deficit is thought to be due to reperfusion injury of a chronically ischemic spinal cord and is referred to as "white cord syndrome" given the pathognomonic finding of hyperintensity on T2-weighted MRI. Three prior cases have been reported. We present a case of transient quadriplegia following posterior cervical decompression.

A 41-year-old male with cervical spondylotic myelopathy presented with bilateral progressive upper extremity weakness, hyperreflexia, and cervical spine MRI showing severe cord compression at C1 and partial hyperintense signal. Intraoperatively, after C1 bony decompression and without perceptible technical cause, the patient experienced a complete loss of both somatosensory evoked potentials (SSEPs) and motor evoked potentials (MEPs) with an eventual return to baseline prior to completing the operation.

The patient awoke from surgery with acute quadriplegia without perceptible technical cause (intraoperative compression or evident anatomic compromise). An immediate postoperative MRI revealed a more pronounced hyperintensity in the central cervical cord on T2-weighted sequences. Treatment with increased mean arterial pressure (MAP) therapy and dexamethasone resulted in the patient regaining some movement over a period of hours and full strength over a period of months.

The mechanism of acute weakness following cervical spine decompression in the absence of perceptible technical cause is not fully understood, but current theory suggests that a reperfusion injury is most likely the cause. It remains a diagnosis of exclusion. Familiarity with this potential postoperative complication can aid in appropriate postoperative therapy with early diagnosis and intervention leading to restored spinal cord function and excellent prognosis.

## Introduction

Cervical spondylosis is the most common cause of myelopathy with an incidence of hospitalizations of 4.04/100,000 in one study [1]. Other causes of cervical myelopathy include ossified posterior longitudinal ligament, trauma, or mass [2]. The clinical course is variable throughout multiple patient groups with some patients responding to conservative management [3]. Many of these patients will undergo surgical decompression for prevention of worsening of symptoms and potential improvement. These symptoms can be attributed to vascular as well as mechanical compressive etiology with direct changes noted in the vascular distribution as well as in the structure of the cord itself [2]. Perioperatively, from cord decompression, patients can occasionally have symptoms of increased myelopathy as well as neurological deterioration in an acute or delayed fashion [4-5]. In some patients, it has been hypothesized that these neurologic symptoms could be from a reperfusion injury [6]. Here, we present a case of a patient that had a severely compressed cervical cord who underwent decompressive surgery providing complete decompression without perceptible iatrogenic technical cord trauma who experienced transient quadriplegia attributed to reperfusion injury immediately after decompression.

Spinal cord reperfusion injury has been reported in the literature but continues to be a rare presentation. On literature review, at least three prior cases have been recognized and reported, two after anterior cervical decompression and one after posterior decompression [7-9]. The anterior decompressions resulted in quadriplegia, and the posterior decompression resulted in hemiplegia. We report the first case of posterior cervical decompression leading to complete albeit transient quadriplegia. The characteristic imaging findings are hyperintense signals on T2-weighted magnetic resonance imaging (MRI). This is thought to be due to reperfusion in a chronically ischemic cord after decompression and has resulted in this condition being referred to as “white cord syndrome.” The purpose of our review is to present a case of transient quadriplegia following posterior cervical decompression and to review the literature regarding this phenomenon. The goal is to educate our colleagues about the potential of this etiology, further understand the pathophysiology, and explore potential future treatment and prevention strategies.

## Case presentation

History and examination

**Figure 1:**
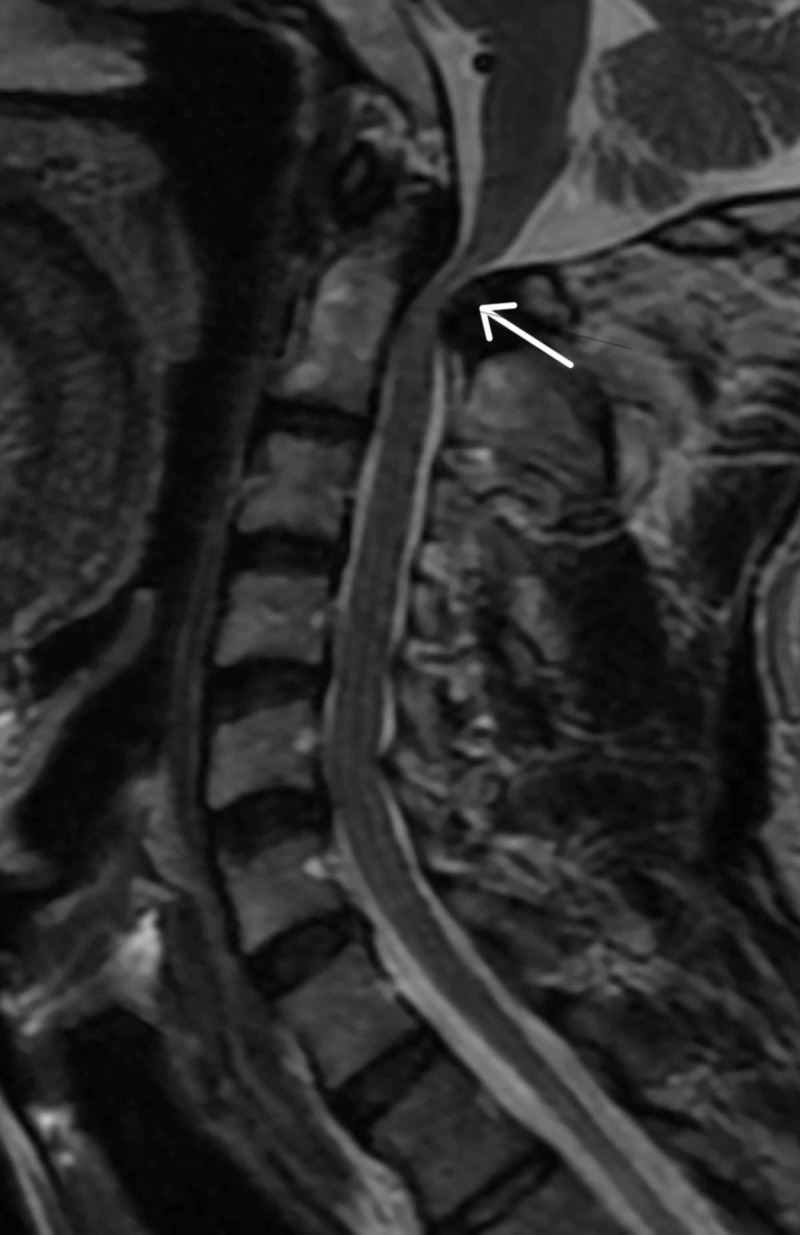
Preoperative T2-weighted MRI of the cervical spine with severe cervical stenosis and cervical myelopathy Preoperative MRI showing severe canal stenosis at C1 with evidence of myelomalacia.

A 41-year-old Hispanic male originally presented to the neurology service for evaluation of low back pain with a burning sensation in bilateral lower extremities. During the exam, it was noted he had brisk reflexes. Therefore, the neurologist recommended a cervical MRI which ultimately demonstrated severe cervical stenosis at C1 with severe cord compression (Figure 1). The patient was referred to the neurosurgery clinic where he endorsed inability to carry things for long periods of time due to hand weakness that had been worsening over six months. Moreover, he stated that he was unable to perform his job as a butcher due to inability to hold his knife. Upon reviewing the patient's MRI and based on clinical exam of hyperreflexia throughout, bilateral clonus, bilateral Hoffman's, and 4+/5 strength in bilateral deltoids and grip, it was quickly determined that the cervical cord decompression was a priority, and he was scheduled for a C1 laminectomy with intraoperative neuromonitoring.

Operation

Prior to positioning as well as intraoperatively, somatosensory evoked potentials (SSEPs) and motor evoked potentials (MEPs) were present, equal, and reproducible in all four extremities. Anesthesia was asked to keep mean arterial pressure (MAP) greater than 85 mmHg throughout the entire surgery. After removal of the posterior C1 arch, all SSEPs and MEPs were still present and reproducible. Shortly thereafter, the neuromonitoring technician was manipulating the needles in the scalp and when asked the reasoning, we were informed that SSEPs were gone from the hands, followed by all extremities, followed by loss of MEPs in the upper extremities. At that time, we stopped all surgical manipulation, and the MAP was confirmed to be greater than 85 mmHg that we requested at the beginning of the case. Nonetheless, we asked to push the MAP greater than 95 mmHg. An intraoperative fluoroscopic lateral image was obtained which confirmed no subluxation or malalignment of the cervical spine that could potentially compress the cord and, therefore, explain the neuromonitoring changes. As an extra precaution, and although there was no cord compression evident at the level of the C2, we cautiously continued to perform a partial laminectomy of superior C2. Within a few minutes, SSEPs and MEPs started to return.

Postoperative course

**Figure 2:**
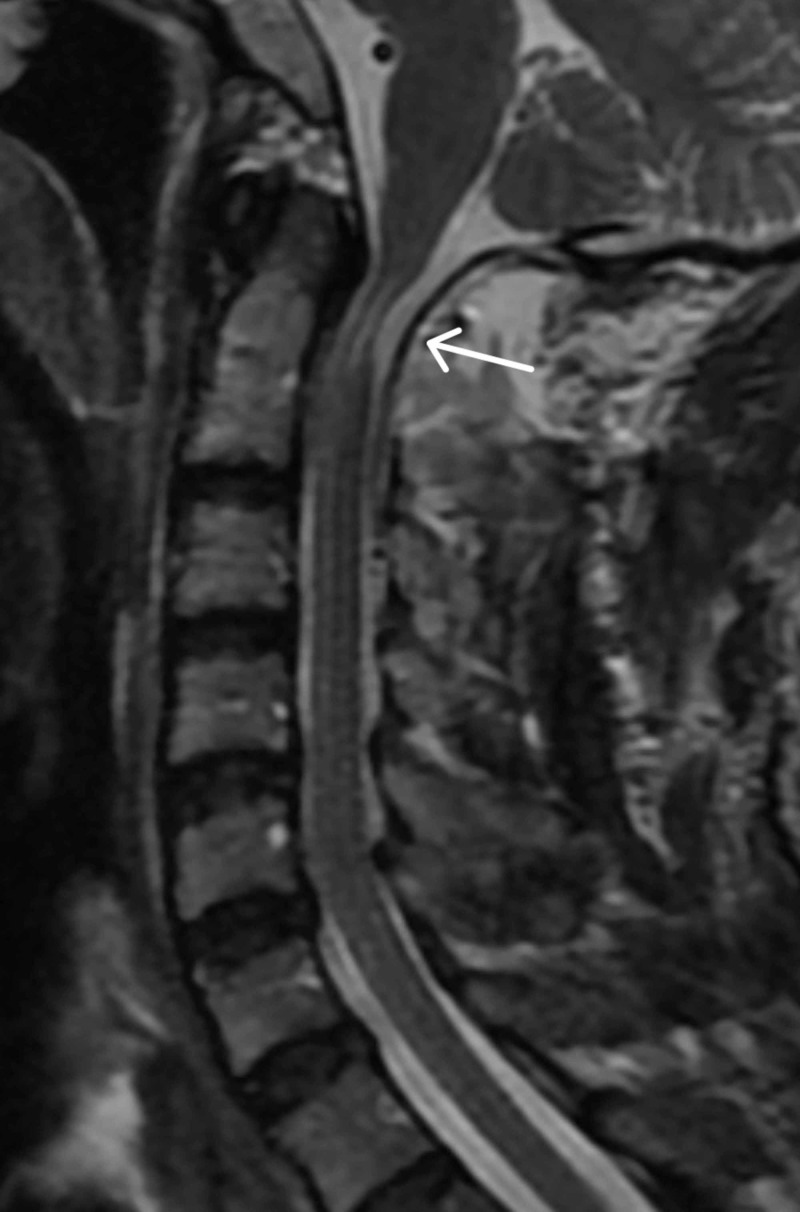
Postoperative T2-weighted MRI of the cervical spine in patient with severe cervical stenosis and cervical myelopathy Postoperative MRI showing complete decompression of cervical spinal cord and hyperintensity at C1.

The patient recovered from anesthesia and, initially, had minimal movement in all four extremities consistent with 1/5 motor strength throughout. Within minutes, he was able to bend his knees bilaterally against gravity and his hand grip became 4/5 bilaterally. The operating room was kept on standby, and the patient was immediately taken to undergo MRI of the cervical spine to rule out cord compression by any mass-occupying lesion such as a hematoma or a migrated tissue fragment. The MRI revealed complete decompression of the cervical spinal cord at the previously compressed level and the absence of any evidence of compression of the entire cervical cord (Figure 2). A cord signal was visible on T2-weighted image, most likely, from the initial severe cord compression which was not visible until the cord re-expanded to some degree. (The continued decrease in cord caliber is an indication of the chronicity of the pathology). We continued to keep the MAP > 90 mmHg for a minimum of five days in the ICU. We re-examined motor strength; the patient was given dexamethasone 10 mg IV every six hours; additionally, he had physical therapy and occupational therapy work daily.

## Discussion

Previous Cases

A literature review revealed several reported cases of paresis in the absence of known intraoperative direct cord injury with an etiology of reperfusion injury (Table 1). The first case describing this phenomenon in the literature as “white cord syndrome” was in a patient with neck pain radiating to the shoulder and to the lower back. During a C4-6 anterior cervical discectomy and fusion (ACDF), after an interbody graft was placed at the C5-6 level, there were diminished MEP signals with no resolution after removal of the interbody. The interbody graft was replaced, and a second interbody graft was placed at C4-5. Given no resolution of diminished MEPs, both interbody grafts were removed, and an anterior plate was placed. Following the procedure, the patient had a C6-level incomplete quadriplegia. The patient had to undergo surgery again for a more extensive decompressive corpectomy at C5 and was given hydrocortisone 100 mg IV intraoperatively. Following the second procedure, the patient was placed in acute inpatient rehabilitation with an acute spinal cord injury steroid protocol. The patient showed some improvement 16 months postoperatively but was still significantly weak [7].

A second case reports a 64-year-old male who underwent ACDF at C3-4 and C5-6 for severe cervical compression: preoperative MRI showed a T2 hyperintensity in the C3-C4 cervical region and disc herniations at C3-4 and C5-6. Despite the absence of any intraoperative complications, the patient was found to have postoperative motor weakness in all four limbs despite only preoperative grip weakness. The postoperative MRI revealed increased hyperintensity at the level of C5-6. High doses of dexamethasone were given, and the patient saw partial improvement of his motor weakness in the rehabilitation unit [8].

The third reported case is also the first case of white cord syndrome following a posterior decompression of the cervical cord, as opposed to following an anterior approach. Similar to previous cases, there was no requirement for further decompression following the loss of MEPs. The patient was managed on high-dose steroids, eventually improving [9].

Cases of transient neurologic deficit after thoracic spine decompression have been reported as well. In three cases of transient neurological deficit, in the absence of direct cord insult, following the decompression of a severely stenotic thoracic spine, two of the patients had neurologic changes of the multimodality intraoperative monitoring findings in the lower extremities. The remaining patient had absent MEPs and SSEPs at baseline throughout the case. Following the decompression, all three had lower extremity deficits that were transient awhich improved within 1-13 months following surgical decompression [10].

**Table 1:** Literature review of cases involving white cord syndrome

Author	Year	Approach	Myelomalacia on preoperative MRI	Postoperative hyperintensity on T2	Treatment
Chin et al.^7^	2013	Anterior	Yes	Yes	High dose steroids and rehabilitation
Giammalva et al.^8^	2017	Anterior	Yes	Yes	High dose steroids and rehabilitation
Antwi et al.^9^	2018	Posterior	Yes	Yes	High dose steroids and rehabilitation

Pathophysiology

Several mechanisms have been purported as the cause of white cord syndrome, and in the absence of direct mechanical cord insult, the differential includes ischemia-reperfusion injury, microthrombi, and altered perfusion due to internal recoil of the spinal architecture following decompression [10]. Areas of the cord that are chronically compressed can become ischemic, possibly from small artery occlusion as hypothesized by Antwi et al. [9]. The most likely and widely reported cause of injury is the ischemia-reperfusion physiology that occurs as a result of a chronically compressed cord expanding to allow the return of blood with subsequent damage, either by direct trauma from blood flow itself or by the oxygen free radicals contained within the newly available blood. Another element is the lipid peroxidation of the neuronal membrane, which has been theorized as a main cause in the “secondary injury-induced degenerative cascade” [11].

Free radicals induce cellular damage to spinal cord axons through oxidative stress [12]. In rats, after decompression of the spinal cord, increased inflammatory markers, tumor necrosis factor α, and interleukin-1 β11 were observed. These inflammatory markers were noted to occur in conjunction with the reperfusion period [13]. In an additional study involving rodents with induced spinal cord compression, rodents with delayed decompression experienced poorer outcomes and notably had increased spinal cord blood flow compared with early decompressed rodents [14]. Additional rodent studies have identified decreased blood flow to compressed cervical segments compared to normal cervical anatomy [15].

Spinal cord blood flow in the rats was evaluated using MRI FLAIR sequencing after six weeks of spinal cord compression and after decompression showing that after six weeks of compression, blood flow in that area of the cord was reduced whereas after decompression there was a significant increase in blood flow. Sections of rat spinal cord were stained for neuronal nuclei and 8-oxoG DNA (a DNA adduct that results from an increase in reactive oxygen species and participates in DNA damage repair) revealing that rats that underwent decompression had higher numbers of 8-oxoG DNA, suggesting a higher amount of reactive oxygen species. Increased blood flow to the cord coupled with the pathophysiological explanation of increased free radical distribution and measured inflammation in the cord is a very valid proposed mechanism for reperfusion injury in the human spinal cord occurring after delayed decompression. The data suggest that reperfusion after surgical decompression induces chronic oxidative damage in neurons - one of the hallmarks of ischemia-reperfusion injury in both human and rodent cervical spondylotic myelopathy (CSM). Additionally, the study illustrates an association between microglia recruitment in the dorsal horns of the lumbar spinal cord and below-level neuropathic pain in CSM [16].

Prevention and Treatment

Experimentation on rats with cervical spondylotic myelopathy was performed in a decompression-only group and a decompression after administration of riluzole group. Riluzole, a sodium glutamate antagonist, has been hypothesized to prevent decompression-mediated ischemia-reperfusion injury. Also, the results of significantly improved forelimb stride length, forepaw initial contact, and regularity index parameters in the riluzole group suggest some level of protection is gained from riluzole administration. In addition, rats treated with riluzole prior to decompression had lower numbers of 8-oxoG DNA than those that received decompression alone, suggesting preservation from this reactive oxygen species damage [16].

The glucosteroid methylprednisolone (MP) has been used in spinal cord injury for its theoretical ability to inhibit lipid peroxidation and directly slow secondary neuronal degeneration in injured cat soleus motor nerves. Hall et al. were able to recreate these effects with the nonsteroidal lipid antioxidant alpha-tocopherol, serving as evidence that the benefit of steroids in spinal cord injury is their ability to inhibit post-traumatic lipid peroxidation [11]. It has been shown that there is a significant recovery in motor function if methylprednisolone therapy is utilized within eight hours of spinal cord injury and continued for 24 hours. Continuation for up to 48 hours has shown to result in even further improvements in neurological function [17]. It should be noted, however, that methylprednisolone use still has no consistent Class I medical evidence [18].

A prospective randomized controlled study of adult CSM patients undergoing decompression surgery sought to investigate whether remote ischemic preconditioning (RIPC) would protect the spinal cord from ischemic injury. The upper limb was subjected to three five-minute cycles of ischemia with interval reperfusion between cycles for five minutes. Neuron-specific enolase, S100B levels, and SEPs were measured in serum at specific time intervals. There was no difference in recorded SEPs between the RIPC group and the control group, but there were significantly reduced serum S100B and neuron-specific enolase levels in the RIPC group. The rate of recovery at seven days, one month, and three months after the surgery was faster in the RIPC group. There was a statistically significant effect of RIPC on the incidence of neurologic complications due to spinal cord ischemia-reperfusion injury after spine surgery [19].

Hydroxysafflor Yellow A (HSYA), historically used for cerebrovascular and cardiovascular disease, was tested on ischemic/reperfusion (I/R) injury of the spinal cord in rabbits - they were assigned into three groups. 48 hours after reperfusion the animal’s spinal cords were analyzed, and the researchers found that HSYA attenuated I/R induced necrosis, alleviated oxidative stress, which was seen as the increase of superoxide dismutase (SOD) and decreased malondialdehyde (MDA) levels, and protected neurons from apoptosis. Extended to human subjects, HSYA may protect human spinal cords from reperfusion injury by alleviating oxidative stress and reducing neuronal apoptosis [20].

Physical therapy is widely reported and universally accepted as a treatment modality for acute or chronic injury and weakness. Patients with any deficit either prior to, or after surgical decompression should be enrolled in immediate postoperative inpatient physical therapy and subsequent outpatient physical therapy as indicated based on a functional level on discharge.

Future treatment strategies

Our efforts lead to four main conclusions. First, this case presentation and literature review is to educate our spine surgeon colleagues that spinal cord reperfusion injury is a valid suggested etiology for plegia after spinal cord decompressive surgery in the absence of iatrogenic technical trauma to the cord. It is also valid given the absence of any postoperative mass-effect producing lesions such as hematoma, graft, or incomplete decompression, as well as an absence of intraoperative hypotension that would lead to cord hypoperfusion. 

Second, we recommend a sequence of intraoperative and postoperative workup to exclude other etiologies for intraoperative neuromonitoring changes. This allows the surgeon to reasonably reach the diagnosis of exclusion of spinal cord reperfusion injury. The sequence starts with stopping all surgical manipulation, confirming MAP is greater than 85 mmHg, obtaining intraoperative fluoroscopic lateral image to confirm no subluxation or malalignment of the cervical spine that could potentially compress the cord, and obtaining an emergent MRI of the spine at the level of interest while the operating room is kept open and on standby to rule out any lesions causing cord compression such as hematoma, graft, tissue fragments, or incomplete decompression. Third, early diagnosis and intervention, as we have demonstrated in this case, restores spinal cord function and leads to a good prognosis.

Fourth, understanding the pathophysiology of such phenomenon has great potential for future targets for management of spinal cord reperfusion injury. Future treatment strategies are areas ripe for further study with many substances showing promise in animal models. Altogether preventing this injury would be especially beneficial - reduction of postoperative morbidity with riluzole, remote ischemic preconditioning, HSYA, or a combination of these and other therapies could reduce or perhaps eliminate the incidence of this pathology. Until further study into the mechanism and prevention of this pathology is completed, the combination of high dose steroids, keeping the MAP greater than 85 mmHg for five days, and postoperative physical therapy should continue to be the mainstay of treatment.

## Conclusions

Our case is the first to report posterior cervical spinal cord decompression leading to complete quadriplegia. In normal physiologic anatomy, the cervical area of the spinal canal provides the largest diameter for the spinal cord to reside and thus is the most difficult area to be compressed. In all three reported cases as well as the case presented from our institution, preoperative MRI suggested some level of myelomalacia, postoperative hyperintense spinal cord signal was present on T2-weighted MRI, and the patients had unexplained neurological deficits after the loss of SSEPs and MEPs intraoperatively. White cord syndrome is described as such, secondary to its characteristic findings on postoperative T2-weighted MRI of intramedullary hyperintensity after decompression of the cervical spine. The intramedullary cord involvement separates this from reported cases of isolated cervical nerve root palsy. Due to cord involvement, white-cord syndrome results in some form of paralysis in one or more limbs (hemiplegia, paraplegia, or tetraplegia). Direct insult to the spinal cord intraoperatively could cause these MRI findings and should always remain a part of the differential diagnosis. However, the cases reported specifically note no known direct trauma to the spinal cord. In our case as well, iatrogenic technical spinal trauma, any type of cord compression, and hypotensive spinal hypoperfusion were all ruled out. This supports spinal cord reperfusion injury as the etiology for postoperative paraplegia given it is a diagnosis of exclusion. In all the other cases mentioned previously in the literature, none had complete resolution of paraplegia, while the patient in our case has experienced this over months, again supporting the etiology of spinal cord reperfusion injury.
